# Estimating and comparing microbial diversity in the presence of sequencing errors

**DOI:** 10.7717/peerj.1634

**Published:** 2016-02-01

**Authors:** Chun-Huo Chiu, Anne Chao

**Affiliations:** Institute of Statistics, National Tsing Hua University, Hsin-Chu, Taiwan

**Keywords:** Extrapolation, Hill numbers, Microbial diversity, Rarefaction, Sample coverage, Standardization, Good–Turing frequency theory, Spurious singleton, Sequencing error

## Abstract

Estimating and comparing microbial diversity are statistically challenging due to limited sampling and possible sequencing errors for low-frequency counts, producing spurious singletons. The inflated singleton count seriously affects statistical analysis and inferences about microbial diversity. Previous statistical approaches to tackle the sequencing errors generally require different parametric assumptions about the sampling model or about the functional form of frequency counts. Different parametric assumptions may lead to drastically different diversity estimates. We focus on nonparametric methods which are universally valid for all parametric assumptions and can be used to compare diversity across communities. We develop here a nonparametric estimator of the true singleton count to replace the spurious singleton count in all methods/approaches. Our estimator of the true singleton count is in terms of the frequency counts of doubletons, tripletons and quadrupletons, provided these three frequency counts are reliable. To quantify microbial alpha diversity for an individual community, we adopt the measure of Hill numbers (effective number of taxa) under a nonparametric framework. Hill numbers, parameterized by an order *q* that determines the measures’ emphasis on rare or common species, include taxa richness (*q* = 0), Shannon diversity (*q* = 1, the exponential of Shannon entropy), and Simpson diversity (*q* = 2, the inverse of Simpson index). A diversity profile which depicts the Hill number as a function of order *q* conveys all information contained in a taxa abundance distribution. Based on the estimated singleton count and the original non-singleton frequency counts, two statistical approaches (non-asymptotic and asymptotic) are developed to compare microbial diversity for multiple communities. (1) A non-asymptotic approach refers to the comparison of estimated diversities of standardized samples with a common finite sample size or sample completeness. This approach aims to compare diversity estimates for equally-large or equally-complete samples; it is based on the seamless rarefaction and extrapolation sampling curves of Hill numbers, specifically for *q* = 0, 1 and 2. (2) An asymptotic approach refers to the comparison of the estimated asymptotic diversity profiles. That is, this approach compares the estimated profiles for complete samples or samples whose size tends to be sufficiently large. It is based on statistical estimation of the true Hill number of any order *q* ≥ 0. In the two approaches, replacing the spurious singleton count by our estimated count, we can greatly remove the positive biases associated with diversity estimates due to spurious singletons and also make fair comparisons across microbial communities, as illustrated in our simulation results and in applying our method to analyze sequencing data from viral metagenomes.

## Introduction

Advances in high-throughput DNA sequencing have opened a novel way to assess highly diverse microbial communities (Sogin et al., 2006; Roesch et al., 2007; Fierer et al., 2008; Turnbaugh & Gordon, 2009). However, the measurement and comparison of microbial diversity are challenging issues due to sampling limitations (Bohannan & Hughes, 2003; Schloss & Handelsman, 2006; Schloss & Handelsman, 2008; Øvreås & Curtis, 2011). These issues become more challenging when sequencing errors generate spurious low frequency counts especially singletons (Quince et al., 2009; Dickie, 2010; Kunin et al., 2010; Quince et al., 2011; Bunge et al., 2012a; Bunge et al., 2012b). In this paper, we use “species” to refer to taxa or operational taxonomic units (OTUs) under a pre-specified percentage of identity of sequences (Schloss & Handelsman, 2005; Schloss & Handelsman, 2008). We also use “individuals” to refer to sequences or any sampling unit.

In macro-ecology, Hill numbers have been increasingly used to quantify species diversity. An Ecology Forum led by Ellison (2010) (and papers that followed it) surprisingly achieved a consensus in the use of Hill numbers as the proper choice of diversity measure, despite intense debates existing in earlier literature regarding this issue. Hill numbers (or the effective number of species) are a mathematically unified family of diversity indices differing among themselves only by an exponent *q* that determines the measure’s sensitivity to species relative abundances. This family includes the three most important diversity measures: species richness (*q* = 0), Shannon diversity (*q* = 1, the exponential of Shannon entropy), and Simpson diversity (*q* = 2, the inverse of Simpson index). See below for its mathematical formula and interpretation. Hill numbers were first used in ecology by MacArthur (1965), developed by Hill (1973), and reintroduced to ecologists by Jost (2006) and Jost (2007). Hill numbers have been extended to incorporate evolutionary history and species traits; see Chao, Chiu & Jost (2014) for a recent review.

Various ecological measures have been applied to quantify the diversity of microbial communities (Hughes et al., 2001; Curtis, Sloan & Scannell, 2002). Hill et al. (2003) reviewed and discussed the suitability of a wide range of ecological diversity measures for use with highly diverse bacterial communities. Members of Hill numbers are also proposed as promising measures for quantifying microbial diversity. For example, Haegeman et al. (2008), Haegeman et al. (2013) and Haegeman et al. (2014) recommended the use of Shannon diversity and Simpson diversity to measure and compare microbial diversity; Doll et al. (2013) suggested using a continuous diversity profile, a plot of Hill numbers as a continuous function of *q* ≥ 0. In this paper, we adopt the general framework of Hill numbers and use continuous profiles to quantify microbial diversity. The diversity profile for *q* ≥ 0 conveys all information contained in a species relative abundance distribution if community parameters (species richness and relative abundances) are known. In practice, however, community parameters are unknown and thus the true diversity must be estimated from sampling data, meaning that statistical methods are required.

In this paper, we propose two statistical approaches (non-asymptotic and asymptotic) to make fair comparisons of microbial diversity across multiple communities. A non-asymptotic approach refers to the comparison of estimated diversities of standardized samples with a common finite sample size or sample completeness (as measured by sample coverage; see below). This approach aims to compare diversity estimates for equally-large or equally-complete samples; it is based on the seamless rarefaction and extrapolation sampling curves of Hill numbers, specifically for *q* = 0, 1 and 2. Traditional sample-size-based rarefaction for species richness has been widely applied in ecology as a standardization method and also suggested by Dickie (2010) for molecular surveys. For species richness, Colwell et al. (2012) proposed an integrated rarefaction and extrapolation sampling curve for standardizing sample size; Chao & Jost (2012) proposed the corresponding curve for standardizing sample completeness. Hill numbers calculated from a sample, like species richness, are an increasing function of sampling effort and thus tend to increase with sample completeness. Chao et al. (2014) generalized previous papers (Chao & Jost, 2012; Colwell et al., 2012) on species richness to the family of Hill numbers and developed two types of standardization methods (sample-size- and sample-coverage-based). The sample-size- and sample-coverage-based integration of rarefaction and extrapolation together represent a unified non-asymptotic and non-parametric framework for estimating diversity and for making statistical inferences based on these estimates. The rarefaction and extrapolation curves for measures of small value of *q* (say, 0 ≤ *q* < 2) heavily depend on the low frequency counts, especially singletons (Chao et al., 2014).

An asymptotic approach refers to the comparison of the estimated asymptotic diversity profiles. That is, this approach compares the estimated profiles for samples with size tending to be sufficiently large or samples with sample completeness tending to unity. It is based on statistical estimation of the true Hill number of any order *q* ≥ 0. This profile is typically generated by substituting species sample proportions into the diversity formula. However, this empirical approach generally underestimates the true profile, because samples usually miss some of the community’s species due to under-sampling. Finding an analytic reduced-bias continuous diversity profile has been a long-standing challenge. Chao & Jost (2015) recently proposed a resolution to obtain a diversity profile estimator, which infers the asymptotic or true diversities. By applying their diversity profile estimator, the negative bias associated with the empirical diversity curve due to undetected species can be greatly reduced. The authors also used real data sets to demonstrate that the empirical and their estimated diversity profiles may give qualitatively different answers when comparing biodiversity surveys. Chao & Jost’s (2015) diversity profile estimator for low values of *q* (0 ≤ *q* < 2) is strongly affected by the low frequency counts. This is mainly because the observed rare species that produce low frequencies carry nearly all the information about the undetected species and play an important role in almost all statistical inferences in diversity estimation.

However, unlike macro-community ecological data, the low frequency counts, especially singletons from high-throughput DNA sequencing, are subject to various types of sequencing errors at different stages of processing (Quince et al., 2009; Huse et al., 2010; Quince et al., 2011). Some sequences may be misclassified as new taxa, and, accordingly, are also misclassified as singletons. Consequently, the observed singletons are greatly inflated and can comprise more than 60% of taxa in a sample (Buee et al., 2009). Since singletons play crucial roles in both asymptotic and non-asymptotic analyses as described above, our suggested approaches will be seriously affected if the inflated singleton count is not adjusted. A wide range of methods have been developed to reduce or correct sequencing errors (Buee et al., 2009; Quince et al., 2011) at the bioinformatics-processing stage. Without knowledge of the sources of measurement errors, statistical sampling-based methods have also recently been proposed to correct the number of spurious singletons and estimate diversity. Bunge et al. (2012a) and Bunge, Willis & Walsh (2014) proposed a parametric mixture model and a method using “left-censored” data; Willis & Bunge (2015) proposed an approach using the ratio of two successive frequency counts. These statistical approaches generally require different parametric assumptions about the sampling models or about the functional form of the ratio of frequency counts. Some of these parametric assumptions may not be reliably tested, and the different parametric assumptions may disallow comparisons across communities.

In this paper, we propose a novel nonparametric approach to estimate the true number of singletons in the presence of sequencing errors. Here we derive a relationship between the expected frequency of singletons and the expected frequencies of doubletons, tripletons and quadrupletons, based on a modified Good–Turing frequency formula originally developed by the founder of modern computer science Alan Turing, and his colleague Good (1953) and Good (2000). Our estimator of singleton count is thus expressed in terms of the observed frequency counts of doubletons, tripletons and quadrupletons, provided these three frequency counts are reliable. Simulation results are reported to demonstrate an important finding about our proposed singleton count estimator. That is, when there are no sequencing errors and sample sizes are reasonably large, our estimator differs from the true singleton count only to a limited extent; when there are sequencing errors, our estimator is substantially lower than the observed singleton count. Therefore, the discrepancy between the estimated and the observed singleton counts can also be used to assess whether or not sequencing errors were present in the observed data.

Throughout the paper, “*adjusted* data/estimators” refer to those with the observed singleton count being replaced by the estimated count (the observed singleton count is discarded), whereas “*original* or *observed* data” refer to the observed data with spurious singletons possibly present. To quantify and compare microbial diversity, here we propose applying both non-asymptotic and asymptotic analyses to the adjusted data whenever the singleton count is uncertain in measurement. That is, for adjusted data, we present seamless sample-size- and coverage-based rarefaction and extrapolation sampling curves of Hill numbers (focusing on measures of *q* = 0, 1, and 2) and a continuous diversity profile estimator. Simulation results based on various taxa abundance distributions are reported to examine the performance of our method and to compare our results with those obtained from a previous ratio-based method (Bunge, Willis & Walsh, 2014; Willis & Bunge, 2015). Sequencing data from viral metagenomes (Allen et al., 2011; Allen et al., 2013) are used for illustration and comparison. The generalization of our methods to phylogenetic diversity is discussed.

## Methods

### Model framework based on Hill numbers

Assume in a community that there are *S* species indexed by 1, 2,…, *S*, where *S* is an unknown parameter. Let *p*_*i*_ be the unknown species relative abundance of the *i*th species or detection probability of the *i*th species in any randomly observed individual, *i* = 1, 2,…, *S*, $\sum\nolimits_{i = 1}^S {{p_i} = 1} $, and *X_i_* be the number of individuals of the *i*th species detected in the sample of size *n*. Let *f_k_* (abundance frequency counts), *k* = 1, 2,…, *n*, be the number of species that are observed exactly *k* times or with *k* individuals in the sample. Here, the unobservable *f*_0_ denotes the number of undetected species in the sample; *f*_1_ denotes the number of singletons and *f*_2_ denotes the number of doubletons observed in the sample.

Given a species relative abundance set {*p*_1_, *p*_2_,…, *p_S_*}, the Hill number of order *q* is defined as:

(1a) $${}^qD = {\left( {\sum\limits_{i = 1}^S {p_i^q} } \right)^{\!\!\!1/(1 - q)}},\quad q \ge 0,\;\;q \ne 1.$$

For all *q* ≥ 0, if ^*q*^*D* = *k*, then the diversity of order *q* of the actual community with relative abundance set {*p*_1_, *p*_2_,…, *p_S_*} is the same as that of an equivalent reference community with *k* equally abundant species (i.e., with relative abundance set {1/*k*, 1/*k*, …, 1/*k*}). This is why Hill numbers are referred to as the effective number of species or as species equivalents. Since the L^p^ norm is widely used in various disciplines, we here provide a very simple and intuitive connection between the L^p^ norm and Hill numbers. Note that the L^p^ norm for the relative abundance set of the actual community is ${(\sum\nolimits_{i = 1}^S {p_i^q} )^{1/q}}$, whereas the corresponding L^p^ norm for the equally abundant reference community is ${(\sum\nolimits_{i = 1}^k {{{(1/k)}^q}} )^{1/q}} = {k^{(1 - q)/q}}$. If the two L^p^ norms are equal, then we have ${(\sum\nolimits_{i = 1}^S {p_i^q} )^{1/q}} = {k^{(1 - q)/q}}$, implying $k = {(\sum\nolimits_{i = 1}^S {p_i^q} )^{1/(1 - q)}}$, which is the formula of the Hill number of order *q* in Eq. (1a).

The parameter *q* determines the sensitivity of the measure to the relative abundance. When *q* = 0, the abundances of individual species do not contribute to the sum in Eq. (1a). Rather, only presences are counted, so that ^0^*D* is simply species richness, which counts *species* equally without regard to their relative abundances. For *q* = 1, Eq. (1a) is undefined, but its limit as *q* tends to 1 is the exponential of the familiar Shannon index, referred to as Shannon diversity (Chao et al., 2014):

(1b) $${}^1D = \;\mathop {\lim }\limits_{q \to 1} {}^qD = \exp \left( { - \sum\limits_{i = 1}^S {p_i^{}\log {p_i}} } \right).$$

The measure for *q* = 1 counts *individuals* equally and thus counts species in proportional to their abundances; the measure ^1^*D* can be interpreted as the effective number of common species in the community. The measure for *q* = 2 discounts all but the dominant species and can be interpreted as the effective number of dominant species in the community. Hill (1973), Tóthmérész (1995), Gotelli & Chao (2013), Doll et al. (2013) and others suggested that biologists should use all the information contained in their data by plotting the diversity as a continuous function of *q* ≥ 0. If the profiles of two communities do not cross, then one of the assemblages is unambiguously more diverse than the other. If they cross, only statements conditional on *q* can be made about their ranking. In most applications, the diversity profiles are plotted for all values (including non-integers) of *q* from 0 to *q* = 3 or 4, beyond which it generally does not change much. Thus, our diversity profile is mainly focused on the range of 0 ≤ *q* ≤ 3.

### Modified Good–Turing frequency formula

The original Good–Turing frequency formula was developed during World War II cryptographic analyses by Alan Turing and I. J. Good. Turing never published the theory but gave permission to Good to publish it. Two influential papers by Good (1953) and Good & Toulmin (1956) presented Turing’s wartime statistical work on the frequency formula and related topics. In an ecological context, the Good–Turing frequency theory answers a question as follows: For those species that appeared *r* times, *r* = 0, 1,…, in a sample of size *n*, how can one estimate the true mean relative abundance *α_r_* of those species? Good and Turing focused on the case of small *r*, i.e., rare species (or rare code elements, in Turing’s case). Mathematically, ${\alpha _r} = \sum\nolimits_{i = 1}^S {{p_i}I({X_i} = r)} /{f_r}$, where *I*(*A*) is the indicator function, i.e., *I*(*A*) = 1 if the event *A* occurs, and 0 otherwise. Ecologists have been using the sample fraction *r*/*n* to infer *α_r_*, but the Good–Turing frequency formula states that *α_r_* should be estimated by r*/*n*, where ${r^*} = (r + 1){f_{r + 1}}/{f_r}$. That is, their estimator is

(2a) $${\tilde \alpha _r} = {{(r + 1)} \over n}{{{f_{r + 1}}} \over {{f_r}}} \equiv {{{r^*}} \over n},\quad r = 0,\;1,...,$$

The above Good–Turing frequency formula has found a wide range of applications in biological sciences, statistics, computer sciences, information sciences, and linguistics, among others. Good (1953) used a Bayesian approach to theoretically justify Eq. (2a) whereas Robbins (1968) derived it as an empirical Bayes estimator. Good (2000) wrote “when preparing my 1953 article, I had forgotten Turing’s somewhat informal proof in 1940 or 1941, which involved cards or urn models in some way, and I worked out a separate proof (Bayes estimator). I still don’t recall Turing’s proof.” Nevertheless, Good (1983, p. 28) provided a very intuitive justification of the Good–Turing frequency formula as follows: Given an original sample of size *n*, consider the probability of the event that the next individual will be a species that had appeared *r* times in the original sample. (Mathematically, this probability is simply $\sum\nolimits_{i = 1}^S {{p_i}I({X_i} = r)} = {\alpha _r}\,{f_r}$). If this event occurs, then the species to which the additional individual belongs must appear *r*+1 times in the enlarged sample of size *n*+1. Since the order in which individuals were sampled is assumed to be irrelevant, the total number of individuals in the enlarged sample of size *n*+1 for those species (that had appeared *r* times in the original sample) is (*r*+1)*f_r_*_+1_. Thus, the probability of the aforementioned event in the enlarged sample of size *n*+1 is (*r*+1)*f_r_*_+1_/(*n*+1) ≈ (*r*+1)*f_r_*_+1_/*n*. Dividing this by the number of such species, *f_r_*, we obtain the mean relative abundance *α_r_* of those species, which is given in Eq. (2a). Chiu et al. (2014) modified the Good–Turing estimator to obtain a more accurate estimator:

(2b) $${\hat \alpha _r} = {{(r + 1){f_{r + 1}}} \over {(n - r){f_r} + (r + 1){f_{r + 1}}}},\quad r = 0,\;1,...\,.$$

This modified formula will be used below in deriving our estimator of the true singleton count.

### Singleton count estimation

An intuitive and basic concept in estimating the number of undetected species is that abundant species (which are certain to be detected in samples) contain almost no information about undetected species richness, whereas rare species (which are likely to be either undetected or infrequently detected) contain almost all the information about undetected species richness. Therefore, most nonparametric estimators of the number of undetected species are based on counts of detected rare species, especially the numbers of singletons and doubletons. Chao (1984) derived a lower bound of undetected species richness in terms of counts of singletons and doubletons; the corresponding lower bound of species richness given below is referred to as the *Chao1 estimator*: (Colwell & Coddington, 1994)

$${\hat S_{Chao1}} = \left\{ {\matrix{ {{\,S_{obs}} + [(n - 1)/n][f_1^2/(2{f_2})],{\rm{ }}\hskip10pt\quad {\rm if}\, {f_2} > 0;} \cr {{S_{obs}} + [(n - 1)/n]{f_1}({f_1} - 1)/2,{\rm{ }}\quad {\rm if}\,{f_2} = 0.} \cr } } \right.$$

Applying a similar concept and derivation, we propose below an estimator of singleton count. Given {*p*_1_, *p*_2_,…,*p_S_*} a general expectation formula for the *k*-th order frequency count is:

(3) $$E({f_k}) = \sum\limits_{i = 1}^S {\left( {\matrix{n \cr k \cr } } \right)p_i^k{{(1 - {p_i})}^{n - k}}},\quad k = 0,\;1,\;...,\;n.$$

Based on this formula, the Cauchy-Schwarz inequality

$$\left( {\sum\limits_{i = 1}^S {p_i^{}{{(1 - {p_i})}^{n - 1}}} } \right)\left( {\sum\limits_{i = 1}^S {p_i^3{{(1 - {p_i})}^{n - 3}}} } \right) \ge {\left( {\sum\limits_{i = 1}^S {p_i^2{{(1 - {p_i})}^{n - 2}}} } \right)^2}$$

leads to

$${{E({f_1})} \over n} \times {{6E({f_3})} \over {n(n - 1)(n - 2)}} \ge {\left( {{{2E({f_2})} \over {n(n - 1)}}} \right)^2},$$

which implies

(4a) $$E({f_1}) \ge {{2(n - 2){{[E({f_2})]}^2}} \over {3(n - 1)E({f_3})}}.$$

Replacing the expectation terms by observed data, we obtain a preliminary lower bound for the true singleton frequency count:

(4b) $${\tilde f_1} = {{2(n - 2){{({f_2})}^2}} \over {3(n - 1){f_3}}}.$$

To obtain a more accurate estimator, we evaluate the magnitude of the bias of the preliminary lower bound in Eq. (4b) as

$$\left| {bias\;({{\tilde f}_1})} \right| \approx E({f_1}) - {{2(n - 2){{[E({f_2})]}^2}} \over {3(n - 1)E({f_3})}}.$$

Using the definition of *α_r_* in the Good–Turing frequency formula, we obtain the following two approximation formulas:

$${{E({f_1})} \over n} = \sum\limits_{i = 1}^S {{{1 - {p_i}} \over {{p_i}}}{{\left( {\matrix{n \cr 2 \cr } } \right)}^{ - 1}}E[I({X_i} = 2)]} \approx {{1 - {\alpha _2}} \over {{\alpha _2}}}{\left( {\matrix{n \cr 2 \cr } } \right)^{ - 1}}E{({f_2})^{}},$$

\eqalign{{{{2E({f_2})}}\over{{n(n - 1)}}} = \sum\limits_{i = 1}^S {\frac{{1 - {p_i}}}\over{{{p_i}}}}{{{\left( \matrix{n \\cr 3} \right)}^{ - 1}}E[I({X_i} = 3)]} \approx {\frac{{1 - {\alpha _3}\over\alpha _3}}{\left(\matrix{ n\\3} \right)^{ - 1}}E({f_3})\].

Substituting the above two approximations into the bias formula, we obtain the magnitude of bias:

$$\left| {bias\;({{\tilde f}_1})} \right| \approx {2 \over {n - 1}}\left( {{{1 - {\alpha _2}} \over {{\alpha _2}}} - {{1 - {\alpha _3}} \over {{\alpha _3}}}} \right)E({f_2}).$$

The right hand side of the above formula will be positive for reasonably large sample sizes, because species that are observed three times in a sample should have a larger mean abundance than that of doubletons (i.e., *α*_3_ is larger than *α*_2_). Applying the modified Good–Turing estimates in Eq. (2b) for *α*_3_ and *α*_2_, we then obtain an estimator of the true number of singletons in terms of (*f*_2_, *f*_3_, *f*_4_) for large sample size *n*:

(5) $${\hat f_1} = {{2f_2^2} \over {3{f_3}}} + 2{f_2}\left( {{{{f_2}} \over {3{f_3}}} - {{{f_3}} \over {4{f_4}}}} \right).$$

When there are spurious singletons, we can adjust the Chao1 estimator (Chao, 1984) by replacing the observed singleton count *f*_1_ with the estimated singleton count ${\hat f_1}$. Then we have the Chao1 estimator of species richness based on the adjusted data if *f*_2_> 0:

(6a) $${\hat S_{adjChao1}} = {S_{obs}} - {f_1} + \hat f_1^{} + {{(n - 1)} \over n}{{\hat f_1^2} \over {2{f_2}}},$$

where *S_obs_* denotes the number of species in the original data. When *f*_2_ = 0, a bias-corrected estimator is suggested:

(6b) $$\hat S_{adjChao1}^* = {S_{obs}} - {f_1} + {\hat f_1} + {{\hat f_1^{}({{\hat f}_1} - 1)} \over {2({f_2} + 1)}}.$$

The variance of the adjusted Chao1 estimator and the corresponding 95% confidence intervals via a log normal transformation can be obtained using similar derivations as those for the classic Chao1 estimator (Chao, 1987).

### Non-asymptotic approach: rarefaction and extrapolation based on adjusted data

It is well known that species richness based on sampling data is highly dependent on sample size and sample completeness (Colwell & Coddington, 1994). Chao et al. (2014) showed that empirical Shannon diversity is moderately dependent and that Simpson diversity is weakly dependent on sample size and inventory completeness. They proposed two standardization methods for Hill numbers to compare non-asymptotic diversities across multiple assemblages as described below. For each type of standardization, we here mainly focus on the three measures of *q* = 0, 1 and 2 based on the adjusted data.

1. Sample-size-based rarefaction and extrapolation up to a maximum size. For each diversity measure, we standardize all samples by estimating diversity for a standard sample size, which can be smaller than an observed sample (traditional rarefaction) or larger than an observed sample (extrapolation). Then we construct for each sample an integrated rarefaction and extrapolation sampling curve as a function of sample size. For species richness, the size can be extrapolated at most to double or triple the minimum observed sample size. For Shannon diversity and Simpson diversity, if data are not too sparse, the extrapolation can be reliably extended to infinity to attain the estimated asymptote given below in Eq. (7).
2. Coverage-based rarefaction and extrapolation up to a maximum coverage. Chao & Jost (2012) proposed standardizing samples by matching their sample completeness, which is measured by *sample coverage*, an objective measure of sample completeness due to Turing and Good (1953) and Good (2000). The sample coverage of a given sample is defined as the fraction of the individuals in an assemblage that belong to the species observed in the sample. Contrary to intuition, sample coverage for the observed sample, rarified samples, and extrapolated samples can be accurately estimated by the observed data themselves. The coverage-based rarefaction and extrapolation curve plots the diversity estimates as a function of sample coverage up to a maximum coverage. For species richness, the maximum coverage is selected as the coverage of the maximum size used in the sample-size-based sampling curve. For Shannon diversity and Simpson diversity, if data are not sparse, the extrapolation can often be extended to the coverage of unity to attain the estimated asymptote given below in Eq. (7).

Chao et al. (2014) introduced a bootstrap method to construct 95% confidence intervals associated with each estimated diversity measure. Generally, for any fixed sample size or any degree of completeness in the comparison, if the 95% confidence intervals do not overlap, then significant differences at a level of 5% among the expected diversities (whether interpolated or extrapolated) are guaranteed. However, overlapped intervals do not guarantee non-significance (Colwell et al., 2012); in this case, data are inconclusive.

The sample-size-based approach plots the estimated diversity as a function of sample size, whereas the corresponding coverage-based approach plots the same diversity with respect to sample coverage. Therefore, the two types of sampling curves can be bridged by a *sample completeness curve*, which shows how the sample coverage varies with sample size and also provides an estimate of the sample size needed to achieve a fixed degree of completeness. This curve and all the rarefaction and extrapolation estimators along with their confidence intervals can be obtained using the R package “iNEXT” which can be also downloaded from Anne Chao’s website at http://chao.stat.nthu.edu.tw/software-download/.

### Asymptotic approach: diversity profile estimation based on adjusted data

The Chao & Jost (2015) diversity profile estimator of *^q^D* (Eq. 1a) based on the adjusted singleton count ${\hat f_1}$ and the original non-singleton frequency counts can be expressed as

(7) $$\scale96%\eqalign{{}^q{\hat D_{adj}}\;\;\; = {\left( {\sum\limits_{k = 0}^{n - 1} {\left( \matrix{q - 1 \cr k \cr} \right)} {{( - 1)}^k}\hat \Delta (k)\hskip2pt +\hskip3pt {{{{\hat f}_1}} \over n}{{(1 - A)}^{ - n + 1}}\left[ {{A^{q - 1}} - \sum\limits_{r = 0}^{n - 1} {\left( \matrix{q - 1 \cr r \cr} \right)} {{(A - 1)}^r}} \right]} \right)^{\hskip-4pt1/(1 - q)}},\;\;\;\;\;\;\;\;\;\;q \ge 0,$$

where $\hat \Delta (0)\quad = \quad1,$

$${\hat \Delta _\,}(k)\;\;\;\;\;\; = \sum\limits_{1 \le {X_i} \le n - k} {{{\left( {\matrix{{n - k - 1} \cr {{X_i} - 1} \cr } } \right)} \over {\left( {\matrix{n \cr {{X_i}} \cr } } \right)}}}\hskip5pt = \hskip3pt\sum\limits_{1 \le j \le n - k} {{{\left( {\matrix{{n - k - 1} \cr {j - 1} \cr } } \right)} \over {\left( {\matrix{n \cr j \cr } } \right)}}{f_j}},\,\;\qquad k\hskip4pt = \hskip3pt1,\hskip3pt2,\hskip3pt...,\hskip3pt\;n - 1,$$

and

\[A\hskip5pt =\hskip5pt \left\{\eqalign{ {2{f_2}/[(n - 1){{\hat f}_1} \hskip2pt+ \hskip2pt2{f_2}],\qquad \quad\hskip33pt {\rm if}\hskip2pt\ {f_2} > 0;}\\{2/[(n - 1)({{\hat f}_1} - 1) \hskip2pt+\hskip2pt 2],\quad\hskip5pt {\rm if}\hskip2pt {f_2}\ \hskip2pt=\hskip2pt\;\ 0,\hskip3pt{{\hat f}_1} \hskip2pt\ne \hskip3pt0;}\\{1,\hskip65pt\qquad \qquad \qquad \quad {\rm if}\hskip2pt\ {f_2}\ \hskip2pt=\ \hskip2pt{{\hat f}_1}\hskip2pt = \hskip3pt 0.}\hskip8pt \right\]

The diversity estimator of order *q* in each profile represents the asymptote in the rarefaction and extrapolation curves described above. To compute the profile estimator in Eq. (7) and the corresponding 95% bootstrap confidence interval, we provide R code (Supplemental Text S1) which is a modified version from the script provided in Chao & Jost (2015). We consider the three special cases of *q* = 0, 1 and 2 below.

For *q* = 0, the estimator in Eq. (7) reduces to the adjusted Chao1 estimator given in Eq. (6a). Thus, it is generally a minimum number of species. For *q* = 1, the estimation of the Shannon diversity from incomplete samples is surprisingly nontrivial and has been extensively discussed in many research fields; see Chao, Wang & Jost (2013) for a review and a low-bias estimator. The estimator (7) for *q* = 1 reduces to their Shannon diversity estimator (given below), which can be compared across communities.

$${}^1{\hat D_{adj}} = \exp \left( {\sum\limits_{1 \le {X_i} \le n - 1} {{{{X_i}} \over n}\left( {\sum\limits_{k = {X_i}}^{n - 1} {{1 \over k}} } \right)} + {{{{\hat f}_1}} \over n}{{(1 - A)}^{ - n + 1}}\left[ { - \log A - \sum\limits_{r = 1}^{n - 1} {{{{{(1 - A)}^r}} \over r}} } \right]} \right).$$

This estimator greatly reduces the negative bias associated with the empirical Shannon diversity. For *q* = 2, the Simpson diversity only counts dominant ones, and dominant species always appear in samples and undetected classes are discounted. Thus the Simpson diversity can often be accurately measured and compared across multiple communities. The estimator (7) for *q* = 2 becomes the nearly unbiased estimator of Simpson diversity (Gotelli & Chao, 2013):

$${}^2{\hat D_{adj}} = {\left( {\sum\limits_{{X_i} \ge 2} {{{{X_i}({X_i} - 1)} \over {n(n - 1)}}} } \right)^{ - 1}}.$$

Notice that singleton count is not involved in the above formula, but the sample size *n* is affected by the adjusted singleton count. Consequently, the effect of spurious singleton count is much less pronounced than that for measures of *q* = 0 and 1.

## Simulation Results

Since both non-asymptotic and asymptotic analyses depend on the quality of the estimated singleton count, it is essential to investigate the performance of the proposed estimator in Eq. (5). We conducted a simulation by generating data from six species abundance distributions with various degrees of heterogeneity in species relative abundances (details are provided in Supplemental Text S2). In each model, we fixed the number of species at *S* = 2,000 to mimic microbial communities. Then for each given model, we considered a range of sample sizes (*n* = 2,000 to 10,000 in an increment of 2,000). The degree of heterogeneity in species relative abundances is quantified by the CV (which is the ratio of the standard deviation over the mean) of species relative abundances. When all species relative abundances are equal, CV = 0. A larger value of CV indicates a higher degree of heterogeneity among species abundances.

**Figure 1:**
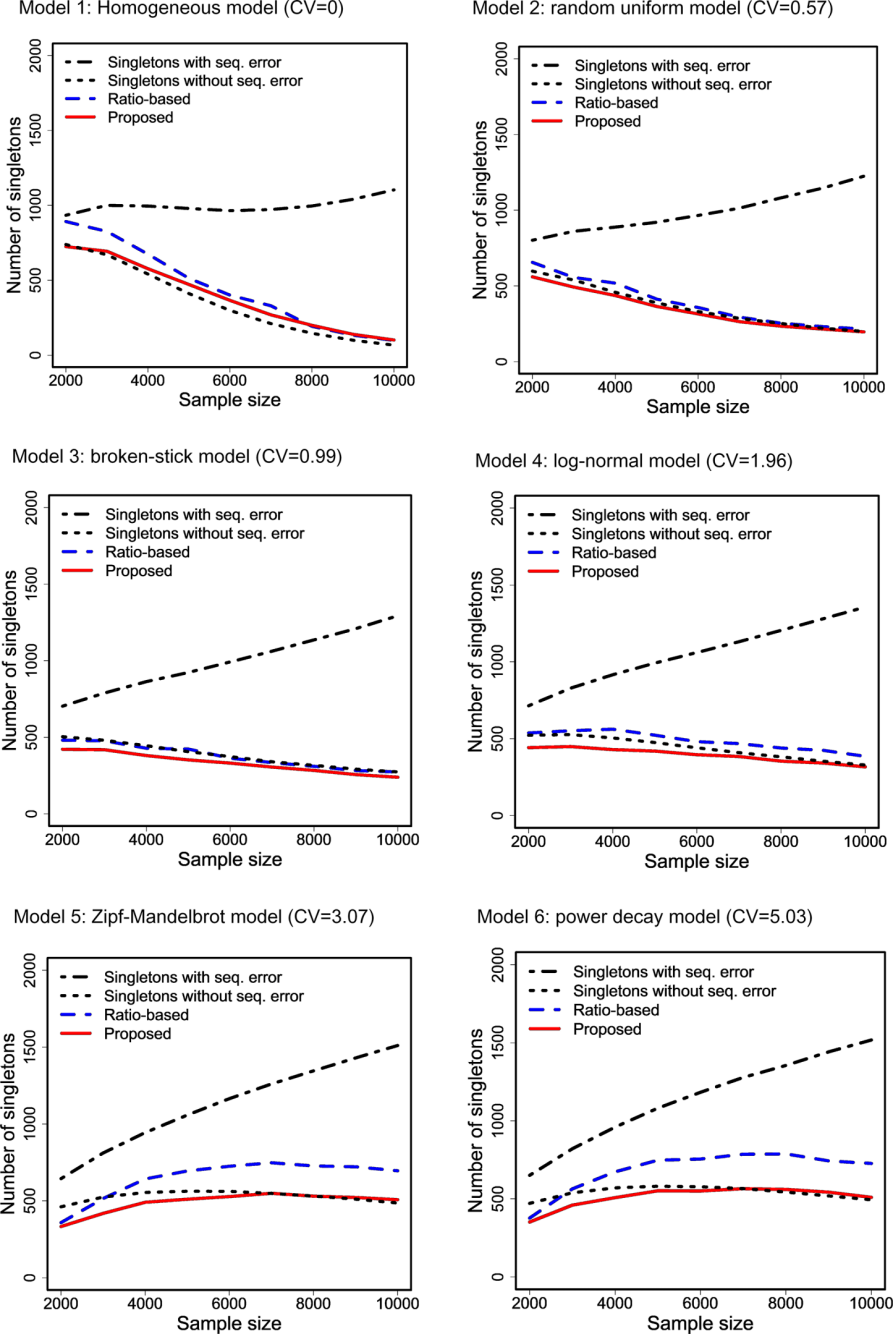
Comparison of the average values of four singleton counts as a function of sample size that was used in data generation. The four singleton counts include the true singleton count generated from the data without sequencing error, the spurious singleton count generated from the data with sequencing error, the adjusted singleton count based on Eq. (5), and the count obtained from the ratio-based method of Bunge, Willis & Walsh (2014) and Willis & Bunge (2015) through the R package “breakaway,” available from CRAN. All values are averaged over 1,000 simulation trials under six species abundance models with various degrees of heterogeneity of the species abundances, as reflected by the CV value (the ratio of the standard deviation over the mean); see Supplemental Text S2 for details.

**Figure 2:**
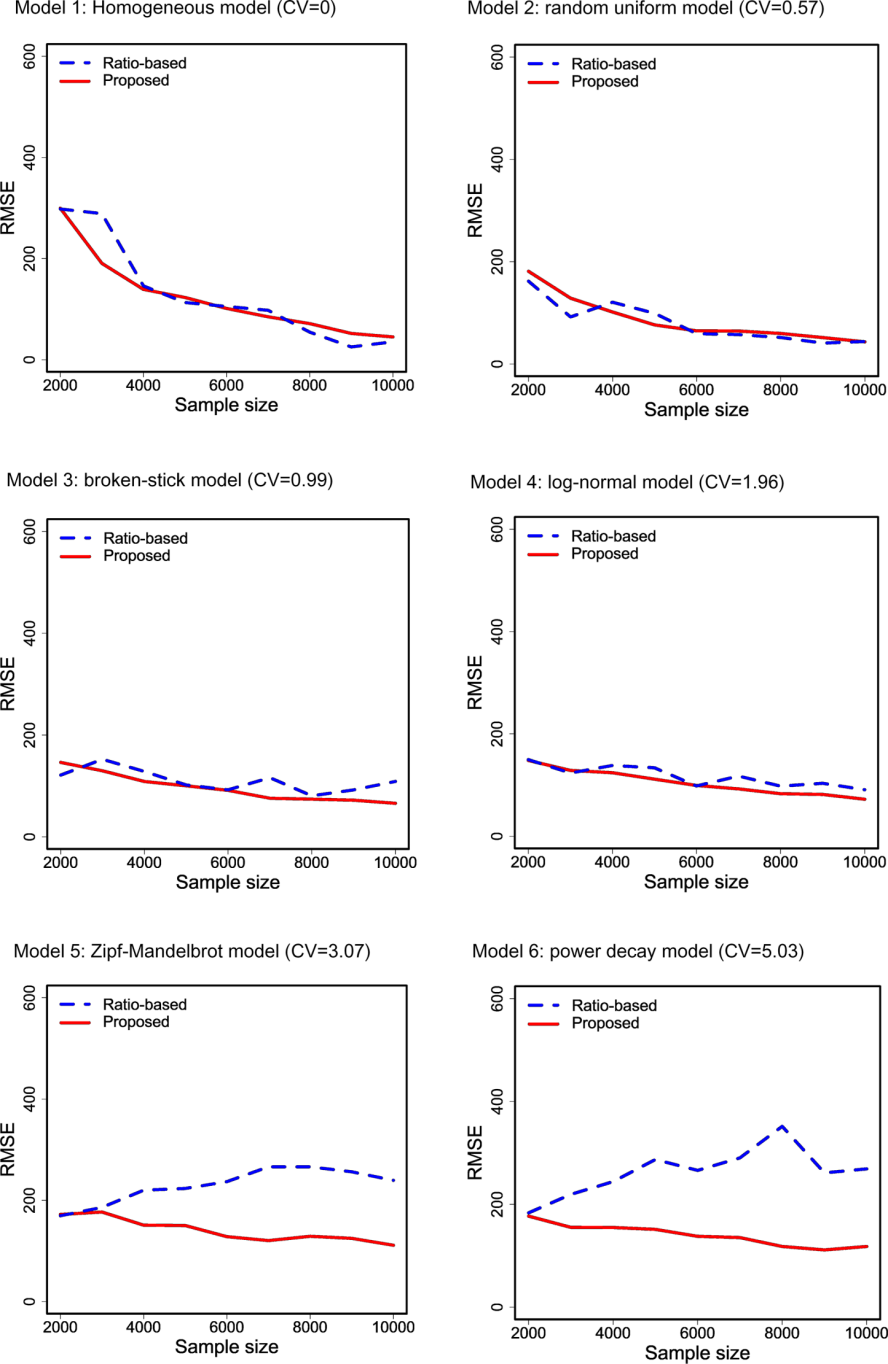
Comparison of the average root mean squared error (RMSE) for two singleton counts (the proposed and ratio-based estimators) as a function of the sample size that was used in data generation. The proposed method is based on Eq. (5), and the results for the ratio method (Bunge, Willis & Walsh, 2014; Willis & Bunge, 2015) were computed using the R package “breakaway.” All values are averaged over 1,000 simulation trials under six species abundance models with various degrees of heterogeneity of the species abundances, as reflected by the CV value (the ratio of the standard deviation over the mean); see Supplemental Text S2 for details.

For each combination of abundance model and sample size, we generated two types of data: (i) data without sequencing errors, and (ii) spurious data with a sequencing error rate of 10%, i.e., there was a 10% chance that a sampled individual was misclassified to a new species and thus became a spurious singleton. In Fig. 1, we show the plots of the average values (over 1,000 simulation trials) of four singleton counts as a function of sample size that was used in data generation. The four singleton counts include the true singleton count generated from the data without sequencing error, the spurious singleton count generated from the data with sequencing error, the adjusted singleton count based on Eq. (5), and the count obtained from the ratio-based method of Bunge, Willis & Walsh (2014) and Willis & Bunge (2015) through the R package “breakaway,” available from CRAN (Comprehensive R Archive Network). In Fig. 2, the corresponding root mean squared errors (RMSEs) for the ratio-based and the proposed methods are shown. The patterns revealed by these plots are summarized below.

Figure 1 reveals that the number of singletons for the data without sequencing error (dotted curve in each panel) generally declines with sample size when sample size becomes sufficiently large, whereas the number of singletons for data with sequencing error (dash-dotted curve in each panel) always increases with sample size, revealing a drastically different pattern; see Dickie (2010) for a similar finding. This pattern can be used to detect whether sequencing error exists in the original data when an empirical accumulation curve for the singleton count can be recorded in the data-collecting procedures.

Simulation results also show that when heterogeneity is low as reflected by low CV values (Model 1 to Model 4) the ratio-based method (dashed curve) and our proposed method (solid curve) yield similar singleton counts that are close to the true data (i.e., data without sequencing error, dotted curve). The RMSEs of the two methods are thus generally comparable (Fig. 2). However, in the highly heterogeneous cases as reflected by relatively high CV values (Model 5 and Model 6), the ratio-method produces much higher singleton counts compared to the true data and thus much larger root mean squared errors than the proposed method, as shown in Fig. 2. In these high-CV cases, our estimator of singleton count still closely matches the true number of singletons, although it exhibits negative bias when sample size is relatively small especially when species abundances are highly heterogeneous.

These simulation results thus imply (i) when there are no sequencing errors (so that the dotted curves represent the singleton counts for data), our estimator differs only to a limited extent from the true data, yielding almost the same diversity inference; (ii) when there are sequencing errors (so that the dash-dotted curves represent the spurious singleton counts for data), our estimator can greatly reduce the raw singleton count and make proper corrections. Therefore, the discrepancy between our proposed estimator of singleton count and the singleton count from the observed data can be used to assess whether sequencing errors were present in data processing. Moreover, this implies that whenever the singletons are uncertain or in doubt, it is worth applying our proposed estimator of singleton count. More simulation results on the effect of spurious singletons on the estimation of asymptotic diversities are provided in Supplemental Text S2; see Discussion.

## Application Results

A number of data sets on frequency counts of contig (contiguous groups of sequences) spectra of viral phage metagenomes from similar or different environments were analyzed in Allen et al. (2013). We select two samples with different environments to illustrate the use of our methods: one sample includes the pooled contig spectra from seven non-medicated swine feces, and the other sample includes the pooled contig spectra from four reclaimed fresh water samples. For simplicity, these two samples/viromes are respectively referred to as “swine feces” sample/virome and “reclaimed water” sample/virome in the following analysis. The frequency counts for the two samples originally provided in the additional file of Allen et al. (2013) are reproduced in Table 1. The empirical diversities and asymptotic analyses are shown in Table 2.

**Table 1:** Frequency counts on contig spectra of phage metagenomic data (Allen et al., 2011; Allen et al., 2013).

Sample	Original *n*	Adj. *n*	*f*_1_	${\hat {\bi f_1}}$	*f*_2_	*f*_3_	*f*_4_	*f*_5_	*f*_6_	*f*_7_	*f*_8_	*f*_9_	*f*_10_	*f*_11_	*f*_12_	*f*_13_
Swine feces	9,988	4,794	8,025	2,831	605	129	41	16	8	4	2	1	1	1	0	0
Reclaimed water	9,973	4,092	7,986	2,105	518	129	50	24	12	7	5	3	2	1	1	1

**Notes:**

Swine feces sample, pooled data from seven swine non-medicated feces; Reclaimed water sample, pooled data from four reclaimed water samples; *f_k_*, number of taxa with *k* sequences in the original data; ${\hat f_1}$, estimated number of singletons based on Eq. (5); Adj. *n*, sample size based on the adjusted data (i.e., the original data with the observed singleton count being replaced by the estimated value).

**Table 2:** Comparison of empirical diversities and estimated diversities (with SE) for the original data, the adjusted data, and two previous methods, based on the phage metagenomics data (Table 1). Previous methods include species richness estimates obtained from CatchAll software (Allen et al., 2013) and from a ratio-based method (Willis & Bunge, 2015). The adjusted data are the original data with the observed singleton count being replaced by the estimated value given in Table 1.

Diversity	Original data		Adjusted data		Previous methods	
	Empirical diversity	Estimated diversity (SE)	Empirical diversity	Estimated diversity (SE)	CatchAll (SE)	Ratio-based method (SE)
**Swine feces sample**						
Species richness (*q* = 0)	8,833	62,057 (1,814)	3,639	10,261 (376)	1,990 (206)	846,113 (249,481)
Shannon diversity (*q* = 1)	8,289	53,835 (1,365)	3,250	9,081 (203)		
Simpson diversity (*q* = 2)	7,348	27,801 (867)	2,742	6,404 (180)		
**Reclaimed water sample**						
Species richness (*q* = 0)	8,739	70,299 (1,973)	2,858	7,134 (273)	1,428 (140)	53,029 (257,637)
Shannon diversity (*q* = 1)	8,066	56,853 (1,451)	2,440	5,849 (130)		
Simpson diversity (*q* = 2)	6,817	21,535 (870)	1,922	3,625 (116)		

In the swine feces original data, there were 8,833 taxa among 9,988 individuals (sequences); the number of singletons was *f*_1_ = 8,025, and the number of doubletons was *f*_2_ = 605. In the reclaimed water data, there were 8,739 taxa among 9,973 individuals, and the first two frequency counts are *f*_1_ = 7,986 and *f*_2_ = 518. In these two original samples, most of the frequencies are concentrated on singletons. Consequently, based on the original data, the Chao1 lower bounds, 62,057 and 70,299 respectively for swine feces and reclaimed water viromes, are greatly inflated due to the presence of spurious singletons. Using Eq. (5), we obtain an estimated singleton count of 2,831 for the swine feces sample, and 2,105 for the reclaimed water sample (Table 1). For each sample, the estimated singleton count is substantially less than the observed singleton count, indicating that sequencing errors were present. The empirical and estimated diversities for the original and adjusted data are shown in Table 2. We also compare in Table 2 our estimates with those based on a ratio-based method (Bunge, Willis & Walsh, 2014; Willis & Bunge, 2015), and with those proposed in Allen et al. (2013) based on the CatchAll software.

From Table 2, as expected, the estimated diversity (species richness, Shannon diversity and Simpson diversity) based on the adjusted data for each sample is much lower than that based on the original data. For species richness, the CatchAll software yields excessively low estimates, even lower than the observed richness of the adjusted data. The ratio-based method, however, yields extremely large estimates for the number of species. In our simulations on species richness as described in Supplemental Text S2, we show that the ratio-based method might severely overestimate the true species richness when the heterogeneity among species abundance is relatively high. The empirical CV values for the swine feces and reclaimed water samples for adjusted date are respectively 0.62 and 0.79. As there are many undetected rare species, the true CV should be much higher than the empirical CV, leading to extremely large species richness estimates for the ratio-based method. All the following analyses are based on our adjusted data, unless otherwise stated.

**Figure 3:**
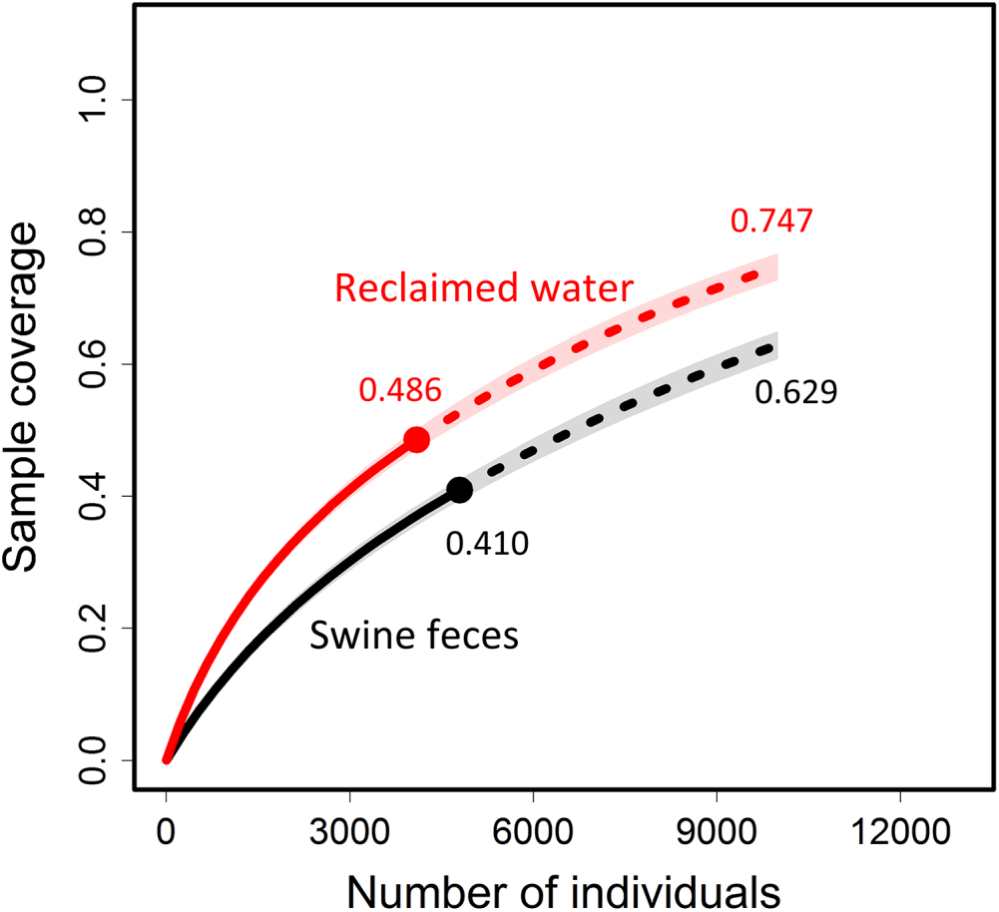
The sample completeness curve based on the adjusted data. Plots of sample coverage for rarefied samples (solid line) and extrapolated samples (dashed line) as a function of the sample size based on the sample frequency counts of contig spectra from seven swine fecal viromes and the sample from four reclaimed fresh water viromes (Allen et al., 2013). Data are given in Table 1. The original singleton count is replaced by the estimated count given in Table 1. The adjusted samples are denoted by solid dots. The 95% confidence intervals (shaded areas) were obtained by a bootstrap method based on 200 replications. Each of the two curves was extrapolated up to 10,000, approximately double the adjusted size of the swine feces sample. The numbers are the sample coverage estimates for the adjusted sample and for the sample of size 10,000.

Before we present the non-asymptotic analyses, we plot in Fig. 3 the sample completeness curve as a function of sample size. The sample completeness of the adjusted swine feces sample is 41%, which is lower than that for the adjusted reclaimed water sample, 48.6%. When the sample size is extrapolated to a size of 10,000 (approximately double the adjusted sample size for swine feces), the coverage of the swine feces sample is increased from 41.0% to 62.9%, whereas the coverage of the reclaimed water sample is increased from 48.6% to 74.7%. For any standardized sample size, Fig. 3 shows that the sample completeness of the swine feces sample is lower than that for the reclaimed water sample of the same size.

**Figure 4:**
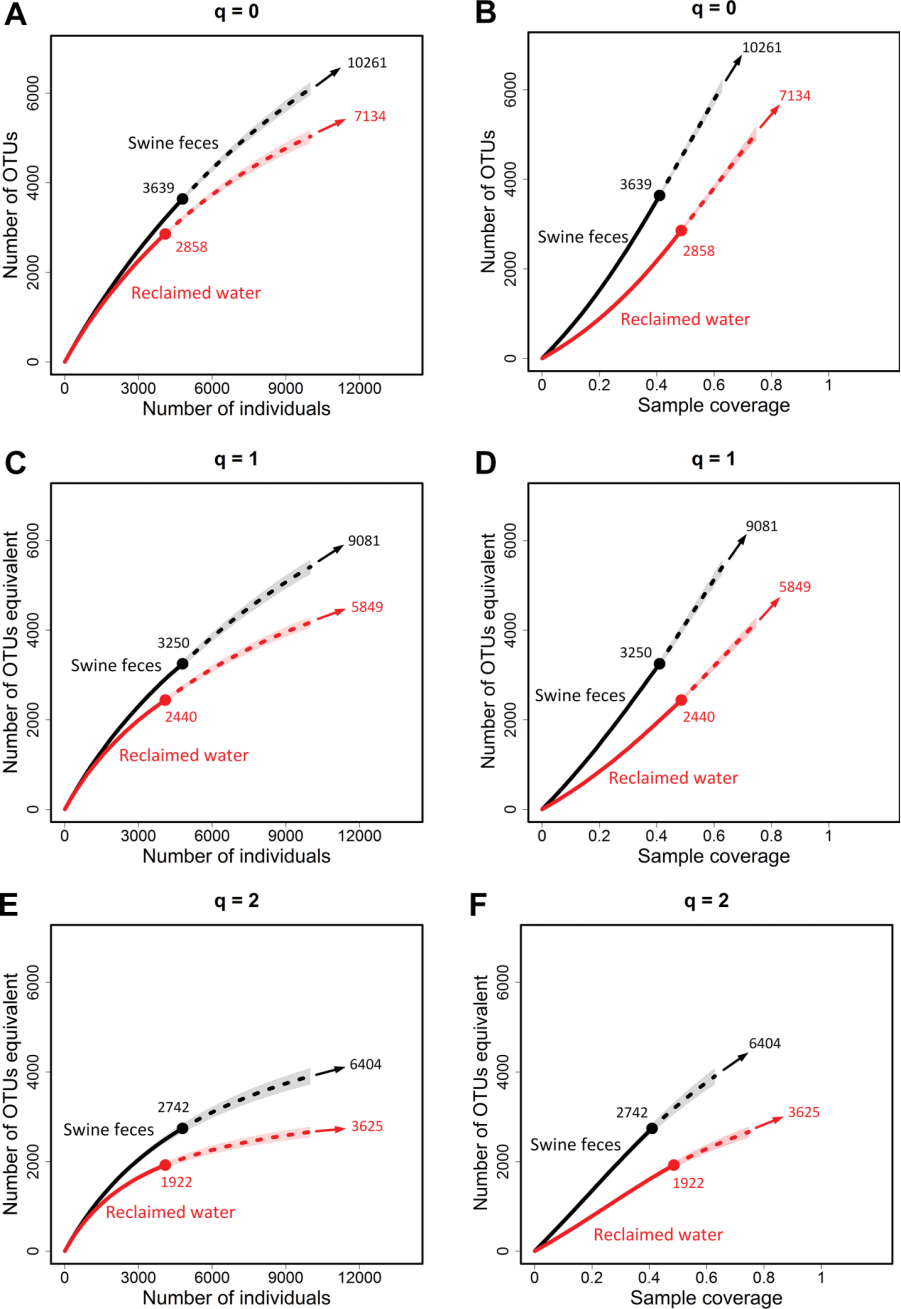
Non-asymptotic analysis: the rarefaction and extrapolation sampling curves based on the adjusted data. Comparison of sample-size-based (A, C, E) and sample-coverage-based (B, D, F) rarefaction and extrapolation for species richness (A, B), Shannon diversity (C, D) and Simpson diversity (E, F) based on the sample frequency counts of contig spectra from seven swine fecal viromes and the sample from four reclaimed fresh water viromes (Allen et al., 2013). Data are given in Table 1. The original singleton count is replaced by the estimated count given in Table 1. The adjusted samples are denoted by solid dots. Rarefied segments are denoted by solid curves and extrapolated segments are denoted by broken curves. Extrapolation is extended up to a maximum size of 10,000. Sample-coverage-based extrapolation is extended to the coverage value of the corresponding maximum sample size (i.e., 62.9% for swine feces viromes and 74.7% for reclaimed water viromes; see Fig. 3). The 95% confidence intervals (shaded areas) are obtained by a bootstrap method based on 200 replications. The estimated asymptotic diversity for each curve is shown next to the arrow at the right-hand end of each curve.

For non-asymptotic analysis, we present in Fig. 4 the sample-size- and coverage-based rarefaction and extrapolation curves along with 95% confidence intervals for three measures: *q* = 0, 1 and 2. The sample-size-based sampling curve is extrapolated up to a maximum size of 10,000, whereas the coverage-based sampling curve is extended up to the coverage of the size 10,000, i.e., the maximum coverage is up to 62.9% for the swine feces sample and 74.7% for the reclaimed water sample.

All plots in Fig. 4 exhibit a consistent pattern, with the diversity curve for the swine feces samples lying above the curve of the reclaimed water sample. In all plots, the 95% confidence intervals for the two samples in any rarefaction/extrapolation curve are disjoint, implying a significant difference. As stated earlier, the extrapolation for Shannon and Simpson diversity, unlike that of species richness, can often be reliably extended to infinity size or complete coverage to reach the asymptotic diversity estimate. Therefore, for Shannon diversity (common taxa richness) and Simpson diversity (dominant taxa richness), the data indicate that the swine feces virome is significantly more diverse than the reclaimed water virome. This is valid not only for the standardized sample size and sample coverage values plotted in Fig. 4, but also for entire viromes. (This is also supported by the asymptotic analysis below). For species richness, the data support this conclusion up to a standardized 62.9% fraction of each virome (Fig. 4B). Beyond that, the data do not provide sufficient information for comparison. This is because the asymptotic species richness estimator is only a lower bound (as opposed to point estimates for the other two asymptotic diversities).

**Figure 5:**
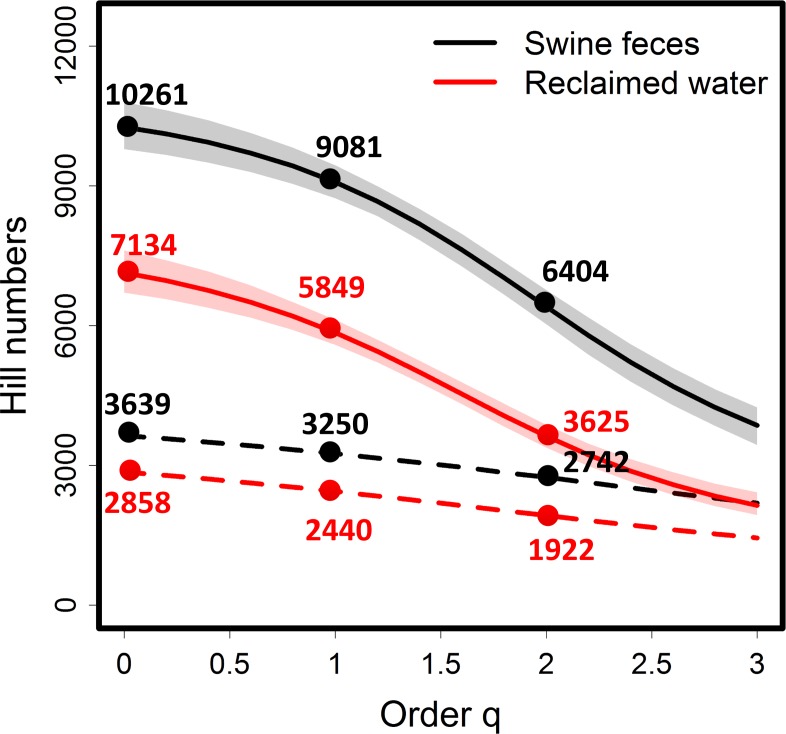
Asymptotic analysis: the asymptotic diversity profile as a function of order *q* based on the adjusted data. The empirical (dashed lines) and estimated (solid lines) diversity profiles for *q* between 0 and 3 based on the sample frequency counts of contig spectra from seven swine fecal viromes and the sample from four reclaimed fresh water viromes (Allen et al., 2013). Data are given in Table 1. The original singleton count is replaced by the estimated count given in Table 1. The plots for the swine feces sample are in black; the plots for the reclaimed water sample are in red. The 95% confidence intervals (shaded areas) are obtained by a bootstrap method based on 200 replications. The numbers (black for swine feces sample, and red for reclaimed water sample) show the empirical and estimated diversities for *q* = 0, 1 and 2.

For the asymptotic analysis, we plot the empirical and estimated asymptotic diversity profiles along with 95% confidence intervals in Fig. 5 when *q* is between 0 and 3. (The empirical and estimated asymptotes of diversities for the special cases of *q* = 0, 1 and 2 are shown in Table 2, and the asymptotic diversity estimates are also shown next to an arrow at the right-hand end of each rarefaction/extrapolation plot in Fig. 5). The empirical diversities (Table 2 and Fig. 5) imply that the two viromes have limited difference in each of the three measures. In contrast, the plots in Fig. 5 reveal that for the asymptotic Shannon diversity, the swine feces virome is substantially more diverse than the reclaimed water virome. A similar conclusion is also valid for the Simpson diversity, confirming our earlier statement in the preceding paragraph.

Table 2 and Fig. 5 show that the adjusted Chao1 estimator in Eq. (6a) gives an estimate of 10,261 taxa for swine feces and 7,134 taxa for reclaimed water virome. Each is five times that obtained from CatchAll (Allen et al., 2013). Since the Chao1 estimate represents only minimum richness, it cannot be used to rank the taxa richness of the two entire viromes. Nevertheless, taxa richness can be compared through the coverage-based non-asymptotic approach, as discussed earlier; see Discussion. By contrast, for diversity of order *q* ≥ 1, we can compare not only the estimated diversities for standardized sample size/completeness but also the estimated asymptotic diversities across communities. In Supplemental Table S1, we also give all the estimated asymptotes of diversities for other data sets provided in Allen et al. (2013).

## Conclusion and Discussion

Whenever the singletons are uncertain or in doubt in sequencing data, it is worth applying our proposed estimator to estimate the singleton count; see Eq. (5). The discrepancy between our estimated singleton count and the observed count can be used to infer whether sequencing errors were present in data processing. Using the estimated number of singleton count and the original non-singleton frequency counts, we can quantify and compare microbial diversity for data sets with different sequencing error rates through non-asymptotic analysis (based on the plots of the sample-size- and coverage-based rarefaction and extrapolation sampling curves) and asymptotic analysis (based on the plot of a continuous asymptotic diversity profile estimator). Illustrative plots for sequencing data from viral metagenomes are shown in Fig. 4 (the non-asymptotic analysis) and Fig. 5 (the asymptotic analysis). Although we have focused on microbial data with spurious singleton counts, both our asymptotic and non-asymptotic approaches are also recommended for analyzing data with reliable singleton counts.

In highly diverse microbial communities, unless strong assumptions or parametric models are made, sampling data often do not provide sufficient information to accurately infer the number of undetected taxa in the sample. Thus, it is statistically infeasible to provide reliable estimates of taxa richness for the entire community. Our estimated species richness (*q* = 0 measure in our asymptotic analysis) theoretically is a lower bound. This implies that fair comparison of asymptotic species richness among multiple communities is not statistically feasible. In this case, fair comparison of taxa richness across multiple assemblages can be made by standardizing sample completeness (i.e., comparing taxa richness for a standardized fraction of population) based on coverage-based rarefaction and extrapolation sampling curves, as illustrated in the real data analysis. By contrast, when the diversity order *q* is away from 0 (say, *q* ≥ 1), rare species have less impact on these diversities, and we generally can infer these diversities up to asymptotes and compare them across communities; see our illustrative example for interpretations. We recommend the use of an estimated diversity profile such as Fig. 5 for asymptotic analysis. If only one or two measures are desired in the inferences of highly diverse microbial diversity, then a perspective from Shannon diversity and Simpson diversity, instead of taxa richness, is more promising and more practical because we can accurately estimate Shannon and Simpson diversity not only for standardized samples but also their asymptotes. Besides, as shown in our simulation results (Figure S1 in Supplemental Text S2), the taxa richness estimator is seriously inflated or affected by spurious singleton counts, whereas the effect on Shannon diversity and Simpson diversity is less serious.

Our proposed estimator of singleton count is in terms of *f*_2_, *f*_3_ and *f*_4_, provided these counts are reliable. A slight generalization of our method can be applied to estimate any frequency count. For example, supposing that singletons and doubletons are both uncertain, we can similarly derive an estimator of doubleton count based on *f*_3_, *f*_4_ and *f*_5_ following exactly the same approach proposed in this paper. Subsequently, Eq. (5) then gives an estimate of singleton count based on the estimated doubleton count, *f*_3_ and *f*_4_. Consequently, our proposed non-asymptotic and asymptotic analyses can be similarly applied to data with the first two frequency counts being replaced by the estimated values. However, the sampling variance of the estimated diversity would be unavoidably increased.

In our approach, the original singleton count is discarded and replaced by our estimated count. In 16S rRNA sequencing or metagenomic sequencing, it is often standard practice to compare sequencing reads against a reference database, such as Greengenes, e.g., see Turnbaugh et al. (2009) or the software MOTHUR (Schloss et al., 2009). The Greengenes alignment tool helps adjust the original singleton count and alleviate the problem of sequencing error. Also, when there are multiple samples, singletons in a given sample have different probabilities of being spurious depending on their total number of reads across samples. Because many local singletons are not global singletons, the cross-sample information may also help adjust the original singleton count. Further investigation examining how to extend our framework to incorporate related covariates (such as cross-sample and database information) is merited.

Finally, we briefly discuss the phylogenetic diversity (PD) because of its broad interest and applications (Martin, 2002; Lozupone & Knight, 2005) in microbial studies. In this paper, all taxa are treated as if they were equally distinct and thus differences among sequences are not considered. Faith’s (1992) PD is the most widely used PD metric to take into account phylogenetic differences among taxa. Faith’s (1992) PD is defined as the total sum of branch lengths of a phylogenetic tree connecting all focal species. Based on sampling data, Chao et al. (2015) recently proposed a non-parametric estimator of the true PD (PD of the entire community, i.e., the observed PD in the sample plus the undetected PD). In the presence of sequencing errors, the inflated singleton count will also affect the estimation of the true PD. Since error-induced singletons will mostly likely fall in a closely related taxon, the effect may not be as pronounced as that in species richness estimation. More investigation is needed to tackle sequencing error and to adjust the Chao et al. (2015) PD estimator. Since Faith’s (1992) PD does not incorporate taxa abundances, Chao, Chiu & Jost (2010) developed a class of abundance-sensitive PD measures which generalize Faith’s (1992) PD to incorporate taxa abundances, and also extend Hill numbers to take into account phylogenetic relationships among taxa. How to extend the proposed analyses presented in this paper (the asymptotic and non-asymptotic analyses) to the class of abundance-sensitive PD is a worthwhile topic of future research.

## Supplemental Information

10.7717/peerj.1634/supp-1 Supplemental Information 1 R codes. R codes for obtaining estimators of Hill numbers.

10.7717/peerj.1634/supp-2 Supplemental Information 2 Simulation results. Simulation results based on six species abundance models.

10.7717/peerj.1634/supp-3 Supplemental Information 3 Diversity analysis. Diversity analyses for the data sets in Allen et al. (2013).

## Additional Information and Declarations

### Data Deposition

The following information was supplied regarding data availability: The data are shown in Table 1 of the main text and in the Supplemental Information.
