# Supplementary material for: Estimating and comparing microbial diversity in the presence of sequencing errors
Source: PeerJ. 2016 Feb 1;4:e1634. doi: 10.7717/peerj.1634 (PMC4741086; doi:10.7717/peerj.1634)
Supplement: Supplemental Information 2 — Simulation results based on six species abundance models. [file peerj-04-1634-s002.pdf]

Supporting Information

Estimating and comparing microbial diversity in the presence of sequencing errors

Chun-Huo Chiu and Anne Chao

Institute of Statistics, National Tsing Hua University, Hsin-Chu, Taiwan, 30043

Supplemental Text S2. **Simulation results based on six species abundance models**

To investigate the performance of the proposed singleton count derived in Equation (5) and the diversity estimator in Equation (7) of the main text, we carried out simulations by generating data sets from various species abundance models. Here we report the results from six representative models. In each model, we fixed the number of species at S = 2000 to mimic the taxa richness of microbial communities.

The functional forms or distributions for species' relative abundances $(p_1, p_2,..., p_S)$ are given below, whereby c is a normalizing constant such that $\sum_{i=1}^{S} p_i = 1$. When species abundances were simulated from a distribution (Models 2, 3 and 4), we first generated a set of 2000 random variables, which we regarded as fixed parameters in the simulation. In each model, we also give the CV (which is the ratio of the standard deviation over the mean) of $(p_1, p_2,..., p_S)$. The CV value quantifies the degree of heterogeneity among the species' relative abundances $(p_1, p_2,..., p_S)$. When all abundances are equal, CV = 0. A larger value of CV indicates a higher degree of heterogeneity among abundances. In the following description, S = 2000 for all models.

Model 1. A homogeneous model with p_i = 1/S and S = 2000. This is the model with no heterogeneity among species relative abundances (CV = 0).

Model 2. A random uniform model with $p_i = ca_i$, where $(p_1, p_2, ..., p_S)$ is a random sample from a uniform (0, 1) distribution. (CV = 0.57).

Model 3. A broken-stick model with $p_i = ca_i$, where $(a_1, a_2, \ldots, a_S)$ is a random sample from an exponential distribution. Equivalently, $(p_1, p_2, ..., p_S)$ follows a Dirichlet distribution with parameter 1 (CV = 0.99).

Model 4. A log-normal model with $p_i = ca_i$, where $(a_1, a_2, \ldots, a_S)$ is a random sample from a log-normal distribution with mean $\mu = 0$, and variance $\sigma^2 = 1$ (CV= 1.96).

Model 5. A Zipf-Mandelbrot model with $p_i = c/(i+5)$, $i = 1, 2, \ldots, S$ (CV = 3.07).

Model 6. A power-decay model with $p_i = c / i^{0.9}$, $i = 1, 2, \ldots, S$ (CV= 5.03).

For each given model, we considered a range of sample sizes (n = 2000 to 10000 in increments of 1000). Then for each combination of abundance model and sample size, 1000 simulated data sets were generated from the abundance model. Two types of data were generated:

(i) Data without sequencing error (i.e., data with the true number of singletons): individuals were randomly selected from a given model and their species identities were correctly recorded.

(ii) Spurious data with a sequencing error rate of 10% (data with spurious singletons): individuals were randomly selected from a given model, but there was a probability of 10% that each sampled individual was misclassified as a new species and thus became a spurious singleton. This was used to mimic the sequencing error with an error rate of 10% for each detected individual to be misclassified as a spurious singleton.

For each model, we display four sub-plots in Supplementary Fig. S1: In Panel (a), we show the plots of the average values of four singleton counts as a function of the sample size that was used in data generation. The four singleton counts include the true singleton count generated from

the data without sequencing error, the spurious singleton count generated from the data with sequencing error, the adjusted singleton count based on Equation (5), and the count obtained from the ratio-based method of Bunge et al. (2014) and Willis & Bunge (2015) through the R package "breakaway", available from CRAN (Comprehensive R Archive Network). All values were averaged over 1000 simulation trials under the six species abundance models. All plots in Panels (a) were also shown in Fig. 1 of the main text; see the main text for the comparisons of the performances of the four singleton counts.

Under each model, Panels (b), (c) and (d) compare the true diversity (Equation 1 in the main text) and the estimated asymptote of diversity (Equation 7 in the main text). There are two estimated diversities, respectively calculated from the spurious data and from the adjusted data. As described in the main text, the "adjusted data" refer to those with the observed singleton count being replaced by the estimated count computed from Equation (5) of the main text.

Panel (b) for each model shows the plots of the true species richness and the average values (over 1000 simulation trails) of the Chao1 estimator for the spurious data, the Chao1 estimator for the adjusted data, as well as the species richness estimator via the ratio-based method described above. It is clear that the Chao1 estimator for the spurious data severely overestimates the true species richness. By contrast, the Chao1 estimator for the adjusted data reduces most of the positive bias and works well for all models, although negative bias exists with the magnitude of the bias increasing with CV value. While the ratio-based method also works when CV value is relatively low (Model 1 to Model 4), the ratio-based species richness estimates exhibit large positive bias when the CV value is relatively high (Model 5 and Model 6).

In Panel (c), we show the plots of the true Shannon diversity and the average values (over 1000 simulation trails) of the estimated Shannon diversity for the spurious data and for the adjusted data. The corresponding plots for Simpson diversity are displayed in Panel (d). Although

the simulation results in Panel (b) of each model demonstrate that the species richness estimation is seriously inflated or affected by spurious singleton counts, the effect on Shannon diversity is moderate and the effect on Simpson diversity is weak, as shown in Panel (c) and Panel (d) in each model). Under each model, both the estimated Shannon and Simpson diversities computed from spurious data overestimate the true diversities, although the bias is not as severe as it is for species richness. Our estimated Shannon and Simpson diversities for the adjusted data exhibit very low bias (when sample size is small) or are nearly unbiased (when sample size is sufficiently large) for all models.

In summary, our estimated asymptotes of diversities presented in Equation (7) of the main text based on the adjusted data greatly remove the positive biases due to spurious singletons. When there are sequencing errors, our procedure always leads to better results; when there are no sequencing errors, our results differ from those based on the true data only to a limited extent. Therefore, our proposed estimator of singleton count can be used to detect the quality of the observed singleton count. This also reveals that whenever singletons are uncertain or in doubt, it is worth applying our estimator of singleton count in diversity analysis and statistical inferences.

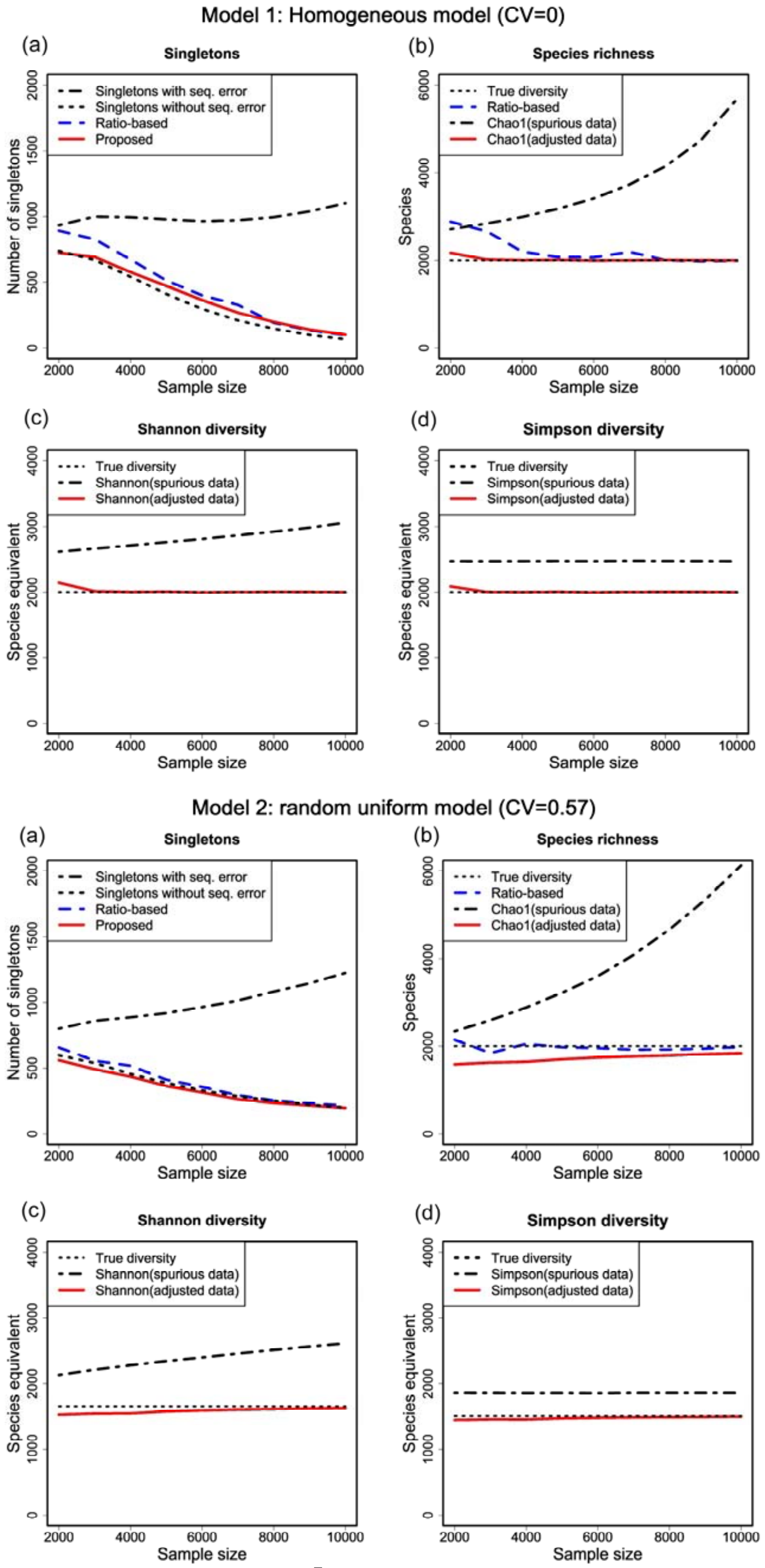
Model 1: Homogeneous model (CV=0)
(a)
Singletons
Singletons with seq. error
Singletons without seq. error
Ratio-based
Proposed
Number of singletons
Sample size
(b)
Species richness
True diversity
Ratio-based
Chao1(spurious data)
Chao1(adjusted data)
Species
Sample size
(c)
Shannon diversity
True diversity
Shannon(spurious data)
Shannon(adjusted data)
Species equivalent
Sample size
(d)
Simpson diversity
True diversity
Simpson(spurious data)
Simpson(adjusted data)
Species equivalent
Sample size
Model 2: random uniform model (CV=0.57)
(a)
Singletons
Singletons with seq. error
Singletons without seq. error
Ratio-based
Proposed
Number of singletons
Sample size
(b)
Species richness
True diversity
Ratio-based
Chao1(spurious data)
Chao1(adjusted data)
Species
Sample size
(c)
Shannon diversity
True diversity
Shannon(spurious data)
Shannon(adjusted data)
Species equivalent
Sample size
(d)
Simpson diversity
True diversity
Simpson(spurious data)
Simpson(adjusted data)
Species equivalent
Sample size

Model 3: broken-stick model (CV=0.99)

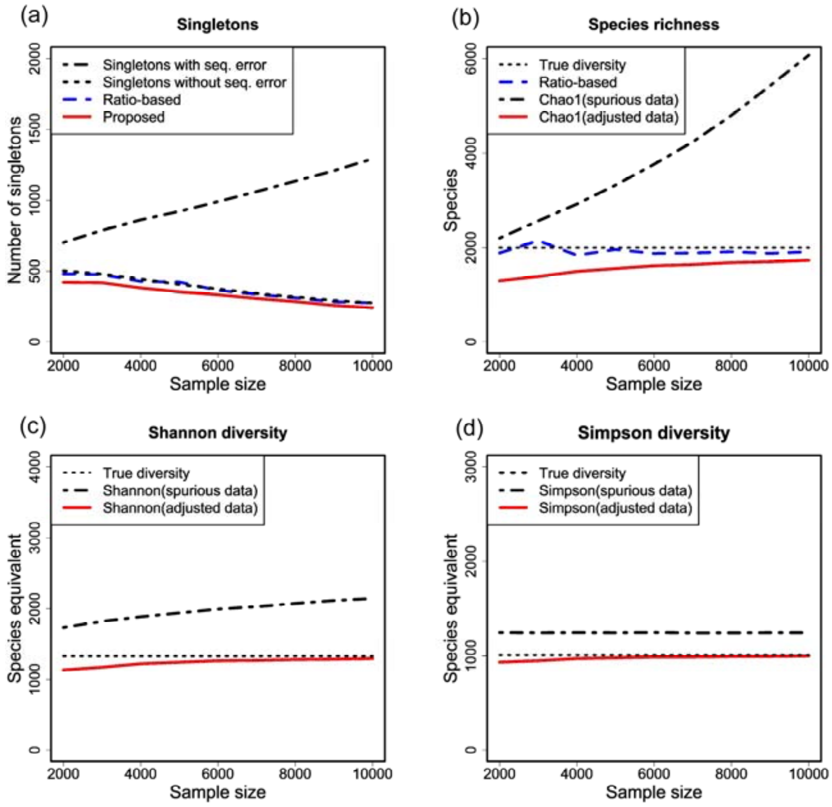

Model 4: log-normal model (CV=1.96)

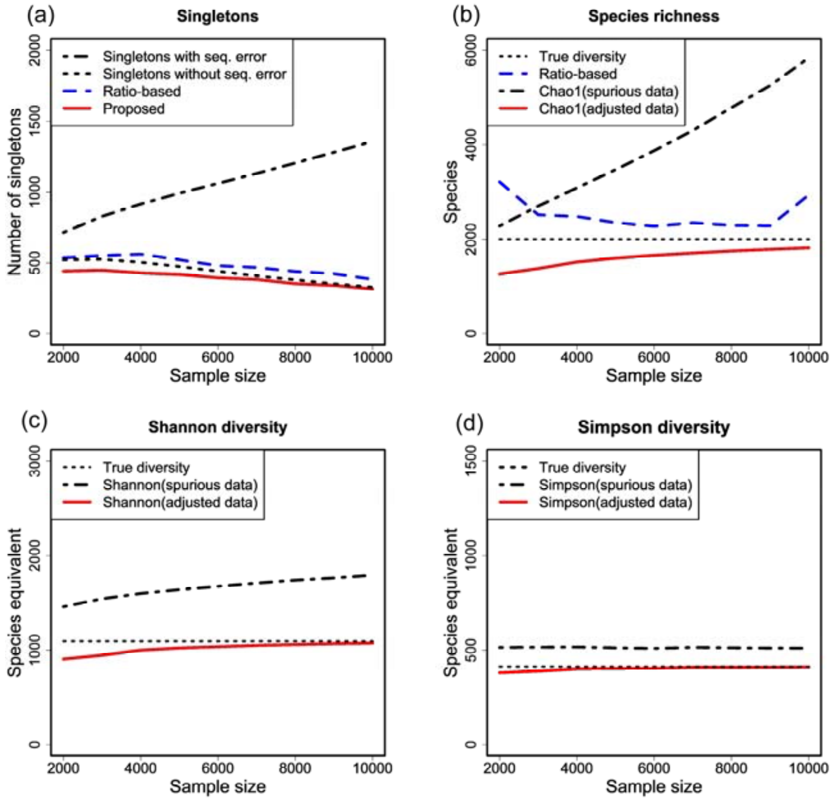

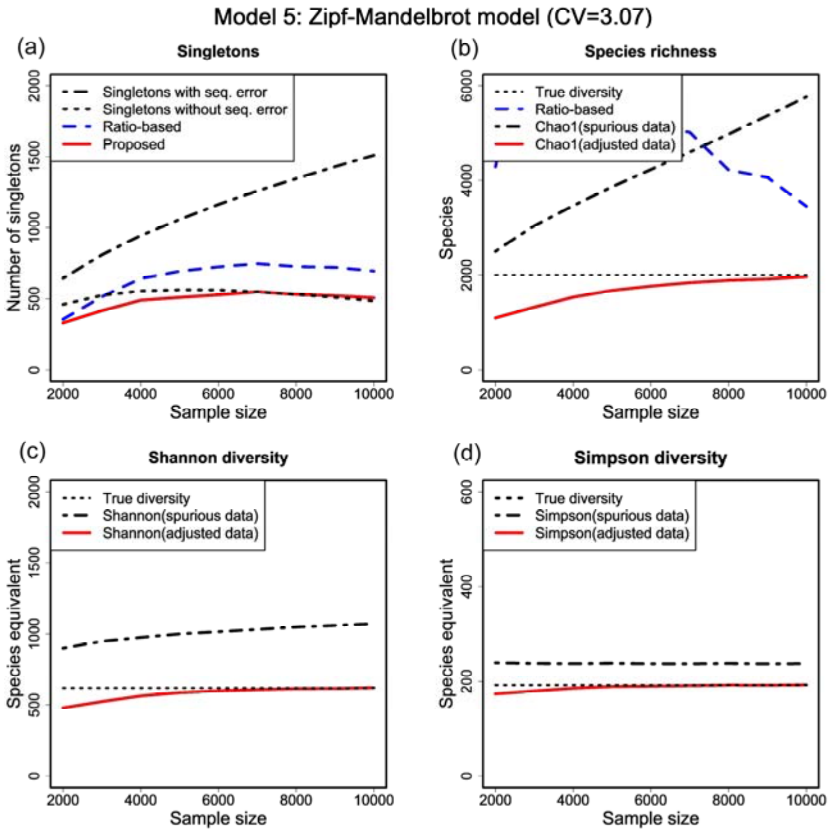
Model 5: Zipf-Mandelbrot model (CV=3.07)
(a)
Singletons
Singletons with seq. error
Singletons without seq. error
Ratio-based
Proposed
Number of singletons
Sample size
(b)
Species richness
True diversity
Ratio-based
Chao1(spurious data)
Chao1(adjusted data)
Species
Sample size
(c)
Shannon diversity
True diversity
Shannon(spurious data)
Shannon(adjusted data)
Species equivalent
Sample size
(d)
Simpson diversity
True diversity
Simpson(spurious data)
Simpson(adjusted data)
Species equivalent
Sample size

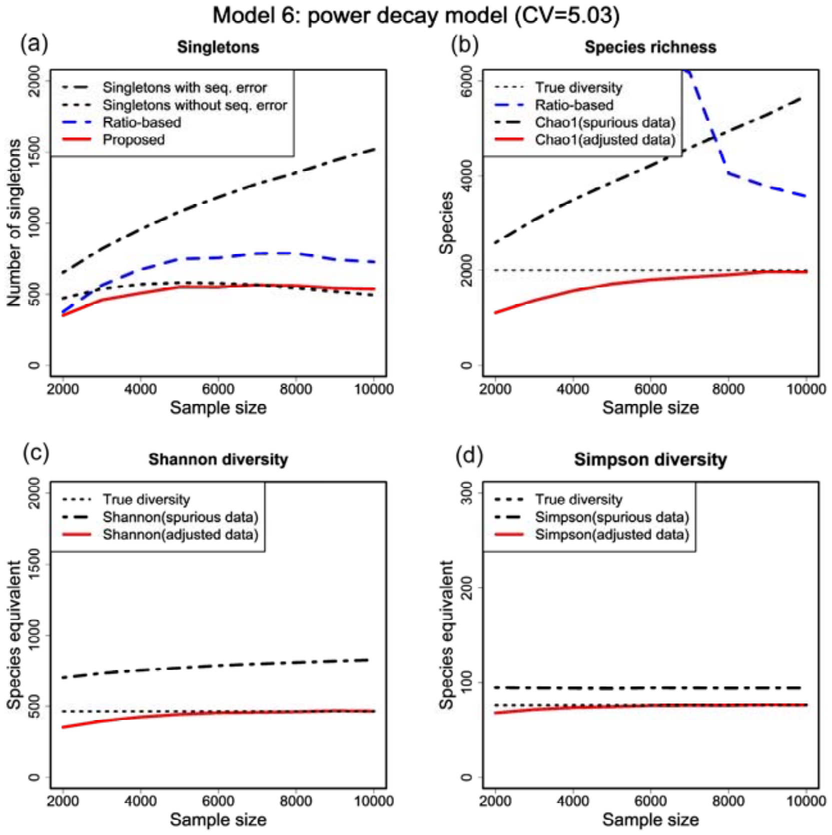
Model 6: power decay model (CV=5.03)
(a)
Singletons
Singletons with seq. error
Singletons without seq. error
Ratio-based
Proposed
Number of singletons
Sample size
(b)
Species richness
True diversity
Ratio-based
Chao1(spurious data)
Chao1(adjusted data)
Species
Sample size
(c)
Shannon diversity
True diversity
Shannon(spurious data)
Shannon(adjusted data)
Species equivalent
Sample size
(d)
Simpson diversity
True diversity
Simpson(spurious data)
Simpson(adjusted data)
Species equivalent
Sample size

Fig S1. Plots of simulation results. Under each model, there are four panels.

Panel (a) compares the average values of four singleton counts: the true singleton count generated from the data without sequencing error, the spurious singleton count generated from the data with sequencing error, the adjusted singleton count based on Equation (5), and the count obtained from the ratio-based method of Bunge et al. (2014) and Willis & Bunge (2015) through the R package “breakaway”, available from CRAN (Comprehensive R Archive Network). All values represent the average values over 1000 simulation trials under six species abundance models.

Panel (b) compares the true species richness, and the average values (over 1000 simulation trails) of the Chao1 estimator for the spurious data, the Chao1 estimator for the adjusted data, and the species richness estimator obtained from the ratio-based approach.

Panel (c) compares the true Shannon diversity and the average values (over 1000 simulation trails) of the estimated Shannon diversity for the spurious data and for the adjusted data.

Panel (d) compares the true Simpson diversity and the average values (over 1000 simulation trails) of the estimated Simpson diversity for the spurious data and for the adjusted data.

Note the scale of the Y-axis in each model may be different in the four panels due to different ranges of diversity.

References

Bunge J, Willis A, Walsh F. 2014. Estimating the number of species in microbial diversity studies. *Annual Review of Statistics and Its Application* 1:427–445. DOI: 10.1146/annurev-statistics-022513-115654.

Willis A, Bunge J. 2015. Estimating diversity via frequency ratios. *Biometrics*, early online version. DOI: 10.1111/biom.12332.
