# Supplementary material for: Estimating and comparing microbial diversity in the presence of sequencing errors
Source: PeerJ. 2016 Feb 1;4:e1634. doi: 10.7717/peerj.1634 (PMC4741086; doi:10.7717/peerj.1634)
Supplement: Supplemental Information 3 — Diversity analyses for the data sets in Allen et al. (2013). [file peerj-04-1634-s003.pdf]

Supporting Information

Estimating and Comparing Microbial Diversity in the Presence of Sequencing Errors

Chun-Huo Chiu and Anne Chao

Institute of Statistics, National Tsing Hua University, Hsin-Chu, Taiwan, 30043

Supplemental Table S1. Diversity analyses for the data sets in Allen et al. (2013)

In the following table, we show diversity analyses for the data sets in Allen et al. (2013)

Orange cells: original data and the Chao1 estimate for the original data;

Yellow cells: empirical taxa richness and estimated asymptotes of diversities for the adjusted data, i.e., data with the original singleton count being replaced by the estimated value computed from Equation (5) of the main text, with SE being obtained by a bootstrap method;

Green cells: taxa richness estimate computed from the software CatchAll (Bunge et al. 2012), and a ratio-based estimate (Bunge et al. 2014, Willis & Bunge 2015) using the function breakaway-nof1 in the R package "breakaway" available from CRAN.

Viromes	Sample	Original sample size	Original empirical taxa richness	Original singleton count f_1	Chao1 for original data	Adjusted empirical taxa richness	Adjusted f_1	Adjusted Chao1 (SE)	Adjusted Shannon diversity (SE)	Adjusted Simpson diversity (SE)	CatchAll (SE)	Ratio-based (SE)
**Swine feces**	Nonmed 21d	9980	7986	6805	37939	3489	2308	6934 (198)	5798 (88)	3969 (89)	2381 (203)	28835 (24374)
	Nonmed 35d	9964	7593	6295	31726	3999	2701	8441 (197)	6595 (111)	4001 (101)	9693 (935)	7754 (701)
	Nonmed38d	9948	6974	5587	26780	3495	2108	6314 (135)	4577 (64)	2611 (66)	4686 (1298)	528777 ($4.7*10^7$)
	Nonmed 63d	9937	6765	5394	26345	3280	1909	5732 (126)	3978 (55)	2207 (52)	5362 (2452)	39540 ($1.6*10^5$)
	Nonmed 77d	10020	7644	6490	37264	3569	2415	7670 (236)	5416 (92)	2631 (84)	5071 (1733)	318464 ($1.6*10^7$)
	Nonmed 85d	9954	8349	7398	50320	3638	2687	9174 (252)	7360 (152)	4274 (137)	1307 (92)	21366 (14234)
	Nonmed 91d	9982	8176	7147	45298	3626	2597	8527 (232)	6701 (127)	3750 (110)	5386 (2052)	7818 (718)
**Human feces**	Infant	477	214	138	521	171	95	316 (35)	165 (11)	90 (8)	94 (30)	201 (105)
	Adult	532	504	482	6957	229	207	1415 (241)	1305 (196)	914 (167)	NA	NA
**Reclaimed fresh water**	Potable	9944	6506	5059	24036	3208	1761	5332 (109)	3767 (55)	2334 (51)	2388 (206)	12624 (20635)
	Effluent	9967	8457	7535	53233	3480	2558	8639 (228)	7088 (137)	4381 (129)	1617 (135)	23492 (34603)
	Nursery	9927	8474	7618	57739	3270	2414	8216 (232)	6550 (132)	3619 (125)	4477 (1652)	20968 ($1.0*10^6$)

	Park	9958	8872	8188	77284	2871	2187	7750 (232)	6433 (151)	3814 (145)	1043 (88)	7768 (651)
**Salt water**	Gulf of Mexico	2500	2359	2297	75640	179	117	369 (43)	244 (20)	118 (15)	103 (37)	451 (110)
	British Columbia	2500	2462	2446	301608	135	119	1029 (291)	660 (150)	174 (39)	NA	NA
	Sargasso Sea	2458	2375	2324	68241	2234	2183	63511 (8007)	58447 (7050)	17510 (3002)	NA	NA
	Arctic	500	474	449	4674	370	345	3273 (514)	3277 (456)	3306 (433)	NA	NA
**Mixed spectra**	Seven swine viromes	9988	8833	8025	62057	3639	2831	10261 (376)	9081 (203)	6404 (180)	1990 (206)	846113 ($2.4*10^6$)
	Four reclaimed water viromes	9973	8739	7986	70299	2858	2105	7134 (273)	5849 (130)	3625 (116)	1428 (140)	53029 (257636)
	Nonmed 85d swine mixed	10002	8963	8243	72465	3286	2566	9438 (296)	8291 (190)	5801 (174)	1958 (235)	10477 (4018)
	Four saltwater viromes	9871	9209	8870	181746	1477	1138	4316 (205)	3057 (112)	1257 (78)	1272 (513)	263149 ($1.0*10^7$)

NA: not available

References

Allen HK, Bunge J, Foster JA, Bayles DO, Stanton TB. 2013. Estimation of viral richness from shotgun metagenomes using a frequency count approach. *Microbiome* 1:5. DOI:10.1186/2049-2618-1-5.

Bunge J, Willis A, Walsh F. 2014. Estimating the number of species in microbial diversity studies. *Annual Review of Statistics and Its Application* 1:427–445. DOI: 10.1146/annurev-statistics-022513-115654.

Bunge J, Woodard L, Böhning D, Foster JA, Connolly S, Allen HK. 2012. Estimating population diversity with CatchAll. *Bioinformatics* 28:1045–1047. DOI: 10.1093/bioinformatics/bts075.

Willis A, Bunge J. 2015. Estimating diversity via frequency ratios. *Biometrics*, early online version. DOI: 10.1111/biom.12332.
